# Diagnostic accuracy of ^18^F Prostate Specific Membrane Antigen (PSMA) PET-CT radiotracers in staging and restaging of high-risk prostate cancer patients and patients with biochemical recurrence: protocol for an overview of reviews

**DOI:** 10.12688/hrbopenres.13801.1

**Published:** 2023-09-20

**Authors:** Andrew Dullea, Lydia O'Sullivan, Marie Carrigan, Susan Ahern, Maeve McGarry, Kirsty O'Brien, Patricia Harrington, Kieran A. Walsh, Susan M. Smith, Máirín Ryan

**Affiliations:** 1 Discipline of Public Health & Primary Care, School of Medicine, The University of Dublin Trinity College, Dublin, Leinster, Ireland; 2 Health Technology Assessment Directorate, Health Information and Quality Authority, Cork, Ireland; 3 Trials Methodology Research Network, College of Medicine, Nursing and Health Sciences, University of Galway, Galway, County Galway, Ireland; 4 School of Pharmacy, University College Cork, Cork, County Cork, Ireland; 5 Department of Pharmacology and Therapeutics, The University of Dublin Trinity College, Dublin, Leinster, Ireland

**Keywords:** Systematic Review, overview of reviews, prostate cancer, justification, PSMA, PET-CT

## Abstract

**Background:** Correct staging and risk stratification is essential in ensuring prostate cancer patients are offered the most appropriate treatment. Interest has been growing in the use of radiotracers targeting prostate specific membrane antigen (PSMA), including the use of ^18^F-PSMA PET-CT, as part of the primary staging or restaging of prostate cancer. Preliminary scoping identified a number of relevant systematic reviews and meta-analyses; however, individually, these each appear to look at only part of the picture. An overview of reviews aims to systematically identify, appraise and synthesise multiple systematic reviews, related to a relevant research question or questions. We present a protocol for an overview of reviews, which aims to collate existing evidence syntheses exploring the diagnostic accuracy of ^18^F-PSMA in staging and restaging of prostate cancer. It also aims to highlight evidence gaps in prostate cancer staging or restaging.

**Methods**: This protocol is reported in line with the Preferred Reporting Items for Systematic Reviews and Meta-Analyses extension for systematic review protocols (PRISMA-P). The search strategy will be designed in consultation with a librarian. Searches will be performed in Medline (EBSCO), Embase (Ovid), Google Scholar and the Cochrane Database for Systematic Reviews, supplemented by a targeted grey literature search, forward citation searching and searching reference lists of included reviews. No language or date restrictions will be applied to the eligibility criteria or the search strategy. Title & abstract and full text screening will be performed independently by two reviewers. Data will be extracted by one reviewer and checked in full by a second reviewer. Quality appraisal will be performed using the Risk of Bias in Systematic Reviews (ROBIS) tool independently by two reviewers, and results will be narratively synthesised.

**Conclusions:** This overview of reviews may be of interest to healthcare professionals, academics and health policy decision-makers.

**Registration:** OSF (September 7, 2023).

## Introduction

Prostate cancer is the second most commonly diagnosed cancer and the fifth leading cause of cancer death among men worldwide ^1^. In Ireland, there are, on average, 3,474 new cases of prostate cancer per 100,000 people each year accounting for, on average, 29.2% of all invasive cancers diagnosed ^2^. The most appropriate treatment strategy for a given patient often depends on the stage of disease, and the risk factors such as prostate specific antigen (PSA) level and Gleason score ^3– 5^. Evidence-based treatment strategies have been developed, and guidelines offer recommendations for specific sub-populations according to stage and risk ^3, 6^. Accurate diagnosis, staging, and risk stratification are therefore essential in ensuring the optimal treatment strategies are offered and the best possible patient outcomes are achieved.

At present, prostate cancer may be detected by digital rectal examinations, PSA levels, and trans-rectal ultrasound (TRUS); histopathological confirmation is usually required to confirm a diagnosis as abnormal findings may be explained by other benign conditions ^5^. However, histopathological confirmation is not always possible or appropriate especially in recurrent or metastatic disease, or populations such as older adults (≥85 years) and those with poor performance status. Metastatic spread to lymph nodes and distant organs is usually detected with ‘conventional imaging’ modalities, including a combination of computed tomography (CT), bone scintigraphy, and magnetic resonance imaging (MRI) ^5^. The **PRO**state **M**RI **I**maging **S**tudy (PROMIS) trial demonstrated that using MRI to triage patients with prostate cancer may allow 27% to avoid primary biopsy and result in an additional 18% clinically significant cancers being detected ^7^.

However, there are a number of persisting limitations with the current reference standard of conventional imaging in the diagnosis and staging of prostate cancer. Despite improvements in the diagnosis and staging of disease with the addition of MRI, the false negative rate for MRI is estimated to be about 6.5% and sensitivity for lymph node imaging remains between 40–73% ^8, 9^. Pooled data from a meta-analysis of patients imaged using MRI also showed a sensitivity of only 57%, 58% and 61% for extra-capsular extension, seminal vesicle involvement and overall stage T3 assessment, respectively ^10^. Similarly, MRI has poor sensitivity in detecting bone metastases. While bone scintigraphy performs better it still has a low sensitivity of approximately 68% for bone metastases ^8^. Hence, there has been a growing interest in radiotracers that may help improve the diagnosis and staging of prostate cancer.

A number of different prostate specific membrane antigen (PSMA)-targeted radiotracers are increasingly being applied in clinical practice in an attempt to improve diagnostic accuracy and the sensitivity and specificity of staging. A number of different manufacturers offer similar PSMA radiotracers that differ slightly in terms of the radioligand attached, the exact antigen, and their pharmacokinetic properties ^11^. In Ireland, ^68^Ga PSMA is used as an alternative to conventional imaging investigations, where available. Such technology takes advantage of the fact that the PSMA protein is rarely expressed in normal prostate tissue, but is highly upregulated and overexpressed in prostate cancer cells and tumour vascular cells ^12^. While some national and international guidelines have adopted and recommended the use of PSMA positron emission tomography (PET) in combination with CT (PET-CT), others have not or have offered weak (as opposed to strong) recommendations ^3, 4, 6, 13^. Most notably, the proPSMA trial found ^68^Ga-PSMA PET-CT to be a suitable replacement for conventional imaging, providing superior accuracy to the combined findings of CT and bone scintigraphy ^14^. One meta-analysis found ^68^Ga-PSMA PET-CT to have a higher sensitivity and a comparable specificity for staging pre-operative lymph node metastases in intermediate- and high-risk prostate cancer compared with MRI ^15^. On a per-patient-based analysis, the sensitivity and specificity of ^68^Ga-PSMA PET-CT were 77% and 97%, respectively, after lymph node dissection at the time of prostatectomy. On a per-lesion based analysis, sensitivity and specificity were 75% and 99%, respectively ^16^. Two retrospective studies also found that ^68^Ga-PSMA PET-CT and PET-MRI demonstrated superiority to MRI alone in the staging of lymph nodes and other areas ^17, 18^. However, the comparison of whole body MRI and PSMA PET-CT in detecting bone metastases has led to inconclusive and conflicting results in two small cohorts ^15, 19^.

Radiolabelling PSMA-targeted agents with ^18^F instead of ^68^Ga may provide several advantages, including improved image resolution and a longer half-life, which may enable better transportation logistics and access to the radiopharmaceutical ^20– 23^. Research into ^18^F-PSMA has accelerated over recent years, yet high quality randomised control trials are lacking. Preliminary scoping of this topic identified a number of relevant systematic reviews and meta-analyses, however individually these each appear to be limited in the scope of their assessment. While some focus on diagnosis, others focus on detection rate, per patient or per lesion sensitivity, and consider a different array of comparators or populations ( *e.g.*, high-risk patients *versus* those with biochemical recurrence, that is, those with a rising PSA after definitive treatment with surgery or radiotherapy). Overviews of reviews use explicit and systematic methods to search for, identify and appraise multiple systematic reviews on related research questions in the same topic area for the purpose of extracting and analysing their results across important outcomes. Thus, the unit of searching, inclusion and data analysis is the systematic review. Overviews often address research questions that are broader in scope than those examined in individual systematic reviews.

We present a protocol for an overview of reviews that aims to collate existing evidence syntheses on a range of diagnostic accuracy measures exploring the use ^18^F-PSMA PET-CT in prostate cancer, using a range of comparators, in distinctly different populations. This overview focuses on high-risk patients and those with biochemical recurrence as these cohorts are most at risk for metastatic disease. In Ireland, these patients currently undergo conventional imaging or where available, ^68^Ga-PSMA PET-CT. ^18^F-PSMA imaging is suggested as a replacement for ^68^Ga-PSMA imaging. While individual systematic reviews may focus on one or more aspects of Tumour, Node, Metastasis (TNM) staging, this overview aims to provide a more comprehensive picture of the evidence for ^18^F-PSMA PET-CT in prostate cancer staging and restaging. It also aims to highlight where evidence gaps exist. This work will help inform the generic justification of this practice in Ireland, and is being carried out by the Health Information and Quality Authority (HIQA).

## Study aims and research questions

This protocol has been registered on the Open Science Framework (September 7, 2023) and developed in line with the Preferred Reporting Items for Systematic Reviews and Meta-Analyses extension for systematic review protocols (PRISMA-P) ^24, 25^. The completed PRISMA-P checklist is available from the registration site. The inclusion and exclusion criteria will follow a Population, Intervention, Comparator and Outcome (PICO) approach.

## Search strategy

Electronic searches will be conducted in Medline (EBSCO), Embase (Ovid), Google Scholar and the Cochrane Database for Systematic Reviews, supplemented by a grey literature search. No language or date restrictions will be applied to the eligibility criteria or the search strategy. The full search strategy can be found on Zenodo as *Extended data* ^26^.

A list of sites included in the grey literature search are given on Open Science Framework as *Extended data* ^24^. Forward citation searching and searching reference lists of included reviews will be conducted to identify other possibly relevant reviews. European Public Assessment Reports (EPARs) for authorised forms of the radiotracer will also be reviewed with a particular focus on identifying possible adverse events not reported within the peer-reviewed literature ^27^.

### Screening

Records will be managed in Endnote V20 ^28^. Following removal of duplicates, title and abstract screening and full-text screening will be completed independently by two reviewers per the eligibility criteria ( Table 1) using Covidence software ^29^. Outcomes may be analysed on a per-patient, per-lymph node, per-lesion, or per-segment basis and similarly outcomes may be analysed according to the effect of PSA levels, Gleason Score, International Society for Urological Pathology (ISUP) grade and other clinicopathological characteristics. No restrictions will be placed on these sub-analyses as they all pertain to TNM staging and the risk category of the population. Studies which only report detection rate, positivity rate or median injection activity will not be included as these variables are of limited value to this overview. No restrictions shall be placed on the method of image analysis – visual, semi-quantitative analysis, or full quantitative analysis using maximum standardised uptake value (SUVmax). No restrictions will be placed on definitions of biochemical recurrence, which may vary depending on the definitive treatment initially offered ( *i.e.*, radical prostatectomy *versus* definitive radiotherapy). Definitions of ‘high-risk’ vary between many organisations and institutions, however any definition will be accepted for the purposes of this overview. No restrictions will be placed on the reference standard, however the ‘gold-standard’ will be considered pathological confirmation determined *via* prostatectomy, pelvic lymph node dissection or biopsy as appropriate to the population under consideration. As study populations in this area often contain both intermediate and high-risk patients, these results will be extracted and will be considered relative to the results from well-defined high-risk populations in other studies.

**Table 1.** Population, Intervention, Comparator, Outcome (PICO).

Patient/Problem:	➣ Adults aged 18 years and older with high-risk prostate cancer undergoing primary staging or adults with biochemically recurrent/persistent prostate cancer undergoing restaging.
Intervention:	➣ ^18^F-PSMA PET-CT used to stage prostate cancer
Comparison:	➣ Reference standards ○ Histopathology ○ Clinical follow up (as defined by the study) ➣ Comparators ○ Conventional imaging using bone scan, CT or MRI. ○ ^68^Ga-PSMA PET-CT
Outcomes:	Any of the following as they relate to TNM staging for prostate cancer: ➣ Sensitivity ➣ Specificity ➣ Accuracy ➣ Negative Predictive Value (NPV) ➣ Positive Predictive Value (PPV) ➣ Positive Likelihood Ratios ➣ Negative Likelihood Ratios ➣ Radiation Dose ➣ Adverse Events ( *e.g.*, hypersensitivity, headache, fatigue, dysgeusia, paraesthesia).
Study Design:	➣ Only systematic reviews and meta-analyses will be considered for inclusion within the overview of reviews. Cochrane defines a systematic review as one that attempts to collate all empirical evidence that fits pre-specified eligibility criteria in order to answer a specific research question ^31^. It uses explicit, systematic methods that are selected with a view to minimising bias, thus providing more reliable findings from which conclusions can be drawn and decisions made ^32, 33^. According to the Cochrane definition, the key characteristics of a systematic review are: ○ a clearly stated set of objectives with pre-defined eligibility criteria for studies; ○ an explicit, reproducible methodology; ○ a systematic search that attempts to identify all studies that would meet the eligibility criteria; ○ an assessment of the validity of the findings of the included studies, for example through the assessment of risk of bias; and ○ a systematic presentation, and synthesis, of the characteristics and findings of the included studies. ➣ Additionally, reviews must have all of the following characteristics: ○ a systematic search of at least two databases. ○ a suitable analysis or subgroup analysis of risk groups or risk factors that allows reviewers to determine the effects on patients with high-risk (or intermediate/high-risk) prostate cancer or those with biochemically recurrent prostate cancer. ○ a quality assessment will also be accepted in lieu of an established risk of bias tool such as QUADAS-2.
Languages:	➣ No language restrictions shall be put in place.

**Key:** ^18^F - Fluorine-18; ^68^Ga – Gallium-68; CT - computed tomography; MRI - magnetic resonance imaging; NPV - negative predictive value; PET-CT - positron emission tomography/computed tomography; PPV - positive predictive value; PSMA – prostate specific membrane antigen; TNM – tumour, nodes, metastasis; QUADAS – Quality Assessment of Diagnostic Accuracy Studies

Disagreements between reviewers will be resolved by discussion or, if necessary, by involving a third reviewer.

## Data collection and analysis

### Data extraction and management

A standardised, electronic data extraction tool will be developed and initially piloted by the reviewers on a minimum of four systematic reviews. The initially proposed data extraction tool is available from the registration site on Open Science Framework ^24^. Data will be extracted by a single reviewer and checked by a second reviewer. Disagreements will be resolved by discussion; any major or systematic disagreements may lead to the involvement of a third reviewer.

In addition to the outcomes listed in Table 1, reviewers will extract information on the number of participants, statistical heterogeneity, assessment of publication biases, exact radiopharmaceutical, reference standard, comparator, and the author, year, and study design of both the reviews and the primary studies they included. Study authors will be contacted for additional information if necessary. If information on sensitivity and specificity is not reported in the systematic reviews, these will be calculated, where possible, using the information on the number of true positives, false positives, true negatives and false negatives.

Data extracted from the primary studies included in the systematic reviews will be cross referenced for discrepant data. Where partial information on a given primary study is reported by more than one systematic review, the record for the primary study will be generated using the information provided across those overlapping systematic reviews.

### Risk of bias assessment

Where reported, the risk of bias assessment of the primary studies included within systematic reviews will be collected by reviewers and presented in the final report. Risk of bias is expected to be generally assessed at the study level, rather than at the level of the outcome for these primary studies. Where systematic reviews contain the same primary study, yet conclude differing levels of bias, the higher of the two biases will be assumed. As part of the inclusion criteria, all reviews should have some form of risk of bias or quality assessment.

The Risk of Bias in Systematic Reviews (ROBIS) tool will be used to assess the risk of bias of the included systematic reviews ^30^. At present, there are no formal guidelines to recommend ROBIS over other tools such as Assessing the methodology quality of systematic reviews (AMSTAR-2) ^31^. Two reviewers will independently assess the risk of bias of all included systematic reviews.

### Data synthesis and certainty of the evidence

Results will be narratively synthesised, as it is anticipated that any meta-analytical approach would be inappropriate due to clinical heterogeneity. The narrative synthesis will be guided by the Synthesis Without Meta-analysis (SWiM) reporting guidelines ^34^. The study design and a summary of the baseline characteristics for all included studies will be presented, followed by the overview’s outcome results, risk of bias of the primary studies and risk of bias of the systematic reviews. It is anticipated that most reviews, while having conducted a risk of bias assessment, will not have ascertained the certainty of the evidence using the Grading of Recommendations, Assessment, Development and Evaluation (GRADE) framework ^35^. In keeping with JBI guidance, reviewers will attempt to apply the principles of GRADE in the overview of reviews to ascertain the certainty of evidence ^36^. A modified version GRADE algorithm for downgrading will be used to generate summary of findings tables ^37^. This approach attempts to map the quality appraisal and risk of bias data onto the GRADE domains as originally set out by Guyatt *et al*. ^38^ The main modifications include the use of ROBIS instead of AMSTAR-2, an explicit focus on clinical heterogeneity rather than just statistical heterogeneity, and changes to the arbitrary sample sizes that determine downgrading (these cut-offs instead will be determined by what the reviewers consider an adequate sample size to determine sensitivity/specificity in these populations).

The overlap of primary studies between systematic reviews will be handled in line with Cochrane guidance ^31^. All reviews may be included if overlap is sufficiently small, however another method such as excluding the older, poorer quality, or less comprehensive reviews may be employed if overlap is significant. A citation matrix will be used to visualise the amount of overlap, and the level of overlap will be determined by calculating the corrected covered area (CCA) ^39^. The CCA is a measure of overlap calculated by dividing the frequency of repeat occurrences of the index publication in other reviews by the product of index publications and reviews, reduced by the number of index publications. A CCA of 0–5 indicates slight overlap, 6–10 moderate overlap, 11–15 high overlap and >15 very high overlap ^40^.

## Deviations from the protocol

Amendments to the protocol prior to and during the conduct of the review will be documented by tabulating version history and important changes in the protocol. Any such deviations will be described in the final report.

## Discussion

This protocol describes each of the steps that will be undertaken as part of this overview of reviews. The proposed overview aims to consolidate existing evidence syntheses that assess different aspects of prostate cancer staging and restaging using ^18^F-PSMA PET-CT to provide a comprehensive overview of the existing evidence, the certainty of the evidence, and the gaps in the evidence at present. We anticipate that the evidence from this overview will be of interest to healthcare professionals, academics and other health policy decision-makers.

HIQA is the competent authority for medical exposures to ionising radiation in Ireland. Part of HIQA’s remit is the justification of new practices before they are generally adopted (also known as Level Two or ‘generic justification’). Generic justification considers whether the benefits of a new practice outweigh the risks, at a population level. In accordance with HIQA’s methods for generic justification, the findings from this overview of reviews will inform HIQA’s decision on whether the practice of ^18^F-PSMA PET-CT should be generically justified ^41^.

## Study status

In progress.

## Data Availability

No data are associated with this article. Open Science Framework: Diagnostic Accuracy of 18F Prostate Specific Membrane Antigen (PSMA) PET-CT radiotracers in staging and restaging of high- risk prostate cancer patients and patients with biochemical failure: Protocol for an Overview of Reviews. https://doi.org/10.17605/OSF.IO/QMEZ5 ^24^ This project contains the following extended data: 18FPSMA_GreyLiteratureSources_OSF_2023_03.docx F18PSMA_DataExtractionTool_OSF_Template_2023_03.xlsx Zenodo: Search strategies for generic justification of 18F-PSMA PET/CT in the staging of primary prostate cancer and the restaging of recurrent and metastatic prostate cancer. https://doi.org/10.5281/zenodo.8159118 ^26^ Repository: PRISMA-P checklist for ‘Diagnostic Accuracy of ^18^F Prostate Specific Membrane Antigen (PSMA) PET-CT radiotracers in staging and restaging of high-risk prostate cancer patients and patients with biochemical recurrence: protocol for an overview of reviews’. https://doi.org/10.17605/OSF.IO/FPZXD ^24^ Data are available under the terms of the Creative Commons Attribution 4.0 International license (CC-BY 4.0).
