# Supplementary material for: Diagnostic accuracy of 18F Prostate Specific Membrane Antigen (PSMA) PET-CT radiotracers in staging and restaging of high-risk prostate cancer patients and patients with biochemical recurrence: protocol for an overview of reviews
Source: HRB Open Res. 2025 Jan 7;6:57. Originally published 2023 Sep 20. [Version 2] doi: 10.12688/hrbopenres.13801.2 (PMC11109553; doi:10.12688/hrbopenres.13801.2)
Supplement: Peer review history

# Reviewer report: Reviewer response for version 1

**DOI:** 10.21956/hrbopenres.15100.r36217

**Published:** 2024-05-15

**Authors:** Laura Evangelista

**Affiliations:** 1 University of Padua, Padua, Italy

The present manuscript aimed to describe the study protocol for evaluating the role of 18F-PSMA in patients with prostate cancer, either in the staging and restaging setting.

 It is clear and concise in the definition of the end-point and in the selection of the population. Also some statistical analysis were mentioned.

 No additional comments from my side.

Is the study design appropriate for the research question?

Yes

Is the rationale for, and objectives of, the study clearly described?

Yes

Are sufficient details of the methods provided to allow replication by others?

Yes

Are the datasets clearly presented in a useable and accessible format?

Yes

Reviewer Expertise:

Nuclear Medicine, Oncology, prostate cancer, breast cancer

I confirm that I have read this submission and believe that I have an appropriate level of expertise to confirm that it is of an acceptable scientific standard.

# Reviewer report: Reviewer response for version 1

**DOI:** 10.21956/hrbopenres.15100.r37847

**Published:** 2024-05-15

**Authors:** Sean Ong

**Affiliations:** 1 The University of Melbourne, Melbourne, Victoria, Australia

The authors present a protocol for a systematic review of reviews in regards to PSMA PET radiotracers and their outcomes. Their reasoning and methods are sound. They have registered their study and have followed PRIMSA guidelines. I have no further comments to add.

Is the study design appropriate for the research question?

Yes

Is the rationale for, and objectives of, the study clearly described?

Yes

Are sufficient details of the methods provided to allow replication by others?

Yes

Are the datasets clearly presented in a useable and accessible format?

Not applicable

Reviewer Expertise:

Urology

I confirm that I have read this submission and believe that I have an appropriate level of expertise to confirm that it is of an acceptable scientific standard.
